# Microtubule‐associated NAV3 regulates invasive phenotypes in glioblastoma cells

**DOI:** 10.1111/bpa.13294

**Published:** 2024-08-03

**Authors:** Aneta Škarková, Markéta Pelantová, Ondřej Tolde, Anna Legátová, Rosana Mateu, Petr Bušek, Elena Garcia‐Borja, Aleksi Šedo, Sandrine Etienne‐Manneville, Daniel Rösel, Jan Brábek

**Affiliations:** ^1^ Laboratory of Cancer Cell Invasion, Department of Cell Biology, BIOCEV, Faculty of Science Charles University Vestec Czech Republic; ^2^ Laboratory of Cancer Cell Biology, Institute of Biochemistry and Experimental Oncology, First Faculty of Medicine Charles University Prague Czech Republic; ^3^ Cell Polarity, Migration and Cancer Unit Université Paris Cité, UMR3691 CNRS, Institut Pasteur Paris France

**Keywords:** amoeboid, glioblastoma, invasion, mesenchymal, NAV3

## Abstract

Glioblastomas are aggressive brain tumors for which effective therapy is still lacking, resulting in dismal survival rates. These tumors display significant phenotypic plasticity, harboring diverse cell populations ranging from tumor core cells to dispersed, highly invasive cells. Neuron navigator 3 (NAV3), a microtubule‐associated protein affecting microtubule growth and dynamics, is downregulated in various cancers, including glioblastoma, and has thus been considered a tumor suppressor. In this study, we challenge this designation and unveil distinct expression patterns of NAV3 across different invasion phenotypes. Using glioblastoma cell lines and patient‐derived glioma stem‐like cell cultures, we disclose an upregulation of NAV3 in invading glioblastoma cells, contrasting with its lower expression in cells residing in tumor spheroid cores. Furthermore, we establish an association between low and high NAV3 expression and the amoeboid and mesenchymal invasive phenotype, respectively, and demonstrate that overexpression of NAV3 directly stimulates glioblastoma invasive behavior in both 2D and 3D environments. Consistently, we observed increased NAV3 expression in cells migrating along blood vessels in mouse xenografts. Overall, our results shed light on the role of NAV3 in glioblastoma invasion, providing insights into this lethal aspect of glioblastoma behavior.

## Graphical abstract

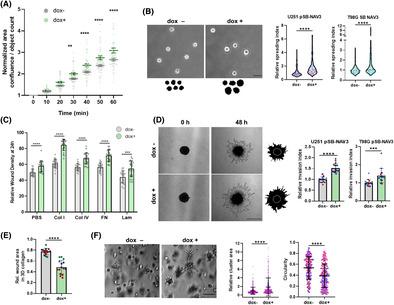

## 1 INTRODUCTION

Cancer cell invasion is a dangerous trait of cancer cells allowing the disease to spread to distant sites. In addition to systemic spread, local invasion can prove lethal when it occurs in regions where cancer cells infiltrate adjacent areas causing damage to critical tissues. This phenomenon is particularly pronounced in the case of brain cancer.

Adult‐type diffuse gliomas are highly heterogenous brain tumors, the most lethal and most common of which are glioblastomas (GBM). Despite multimodal treatment including surgical resection, radiotherapy, and chemotherapy, GBMs are generally incurable. Although less than 2% of GBM metastasize beyond the brain [1], GBM cells are highly invasive. GBM infiltration into surrounding healthy tissue not only renders complete surgical resection of the tumor impossible, but also contributes to treatment failure since infiltrating cells are often more resistant to therapy [2]. GBM dissemination is therefore currently considered a larger threat than the mass effect caused by tumor growth [3], and is the leading contributor to a dismally low median survival of patients diagnosed with GBM of 15 months [4], with a 3‐year survival rate of only 10.5% [5]. Understanding GBM invasion is thus imperative for improving its poor prognosis.

GBM most commonly invade the perivascular niche or migrate along white matter tracts, or alternatively, they can directly disseminate through the brain parenchyma [6, 7]. These invasion routes largely differ in extracellular matrix (ECM) composition and biophysical characteristics [8, 9, 10]. To enable these diverse forms of invasion, GBM cells can adopt different invasion modes, a phenomenon called invasion plasticity. The two main types of invasive modes include protease‐dependent mesenchymal migration, characterized by an elongated shape with protrusions, and protease‐independent amoeboid migration, manifested by round cells undergoing dynamic reshaping and membrane blebbing [11, 12]. In areas rich in hyaluronic acid, diffusely infiltrating GBM cells employ the amoeboid migration mode [13]. Conversely, cells migrating along vessels exhibit characteristics typical of the mesenchymal phenotype, such as an elongated shape facilitated by the formation of cell protrusions and adhesions [7].

Neuron navigator proteins 1‐3 (NAV1‐3) constitute a family of microtubule (MT) associated proteins that participate in the regulation of MT dynamics and reorganization of the cytoskeleton. Their binding to MT plus ends promotes MT extension and stabilizes the polarized growth of protrusions [14]. Moreover, NAV1 was shown to enhance actin polymerization via TRIO and Rac1 [15, 16]. Abundantly present in healthy brain tissue, NAV1‐3 actively contributes to proper axon guidance and neurite outgrowth, playing a significant role in brain development [17]. Notably, specifically neuron navigator 3 (NAV3) is frequently subject to mutations or deletions in various tumors, including those affecting the brain [18].

Given the significance of MTs in cellular migration [19] and particularly in normal and tumoral glial cell migration [20, 21] and the established physiological role of NAV3 in brain tissue, our investigation focused on the specific role of NAV3 in GBM cell migratory behavior.

## 2 RESULTS

### 2.1 Heterogeneity of NAV3 expression in glioblastoma

**FIGURE 1:**
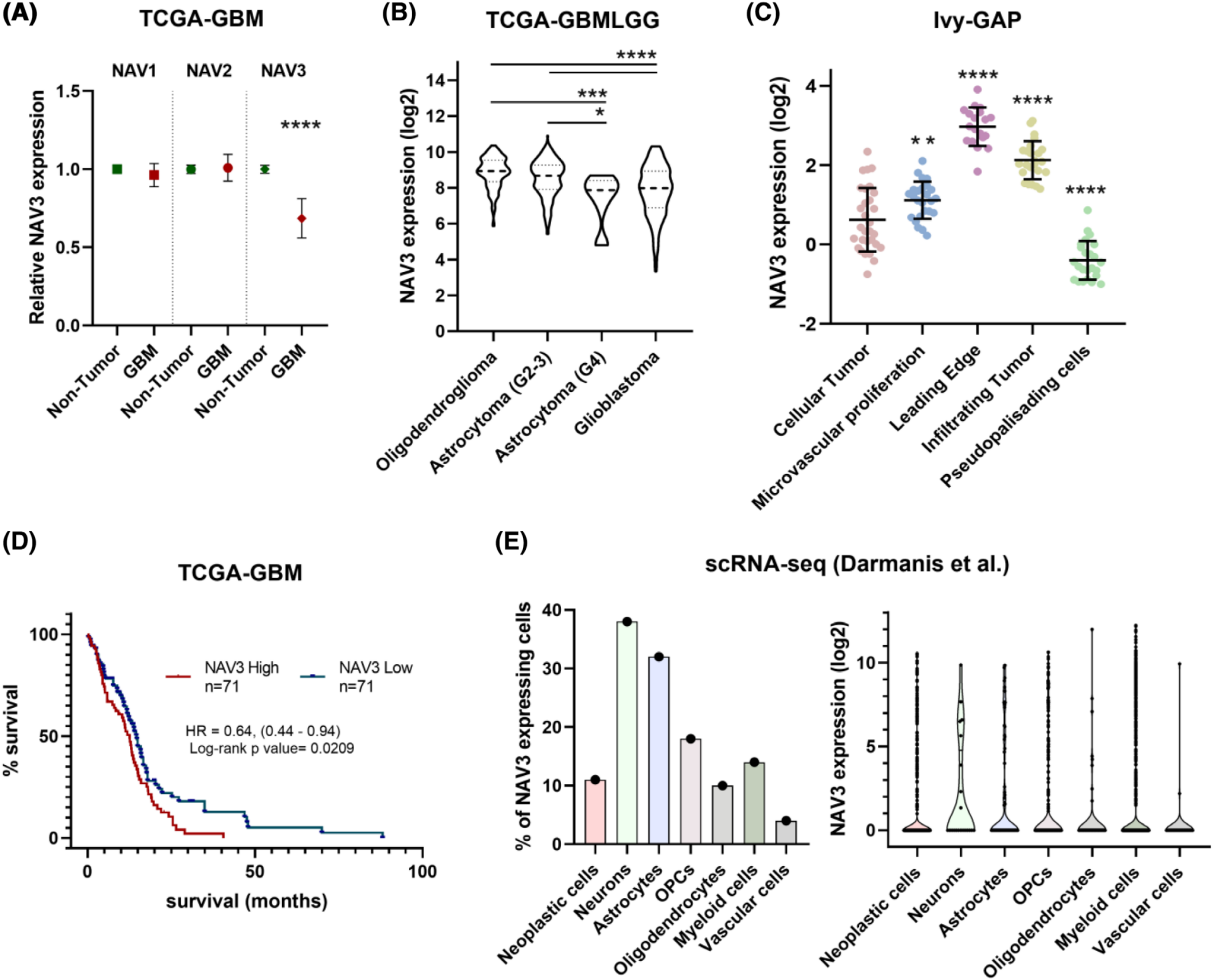
Expression heterogeneity of NAV3 in glioblastoma. Adult‐type diffuse glioma expression datasets were downloaded from GlioVis online platform (http://gliovis.bioinfo.cnio.es/). (A) TCGA expression data of NAV1, NAV2, and NAV3 in non‐tumor brain tissue compared to primary, IDHwt GBM. Statistical significance was determined using *t*‐tests. (B) NAV3 expression levels in TCGA‐GBMLGG dataset reclassified according to the current WHO 2021 classification into the following categories: Oligodendroglioma (IDHmut, 1p19q co‐deleted, grade 2 or 3; astrocytoma, IDHmut (grade 2 or 3); astrocytoma, IDHmut, grade 4, and GBM, IDHwt (glioblastoma histology and/or chromosome 7 gain/chromosome 10 loss and/or TERT promoter mutation). Statistical significance was determined using one‐way ANOVA with post‐hoc Tukey test. (C) Expression values of NAV3 in defined anatomic regions of GBM tissue according to the Ivy GAP dataset. Statistical significance was determined relative to Cellular Tumor using one‐way ANOVA with Dunnett's multiple comparisons test. (D) Survival plot for primary IDHwt GBM according to low/high NAV3 expression. (E) NAV3 expression in individual cell types in GBM as determined by single‐cell RNAseq (Darmanis et al. [23]), data were downloaded from http://gbmseq.org on Novemebr 16, 2021. The percentage of cells expressing NAV3 (left) and the level of NAV3 expression is shown (right). Statistics and visualization of data were performed using GraphPad Prism. Data in (A)–(C) are shown as mean ± SD; *p ≤ 0.05, ** *p* ≤ 0.01, ***p ≤ 0.001, ****p ≤ 0.0001. For more information, see Supplementary Tables.

To assess NAV3 expression in adult‐type diffuse gliomas, we harnessed publicly accessible datasets using the Gliovis online platform [22] and examined NAV3 expression profiles. Analysis of TCGA expression data revealed a significantly lower expression of NAV3 in GBM compared to non‐tumorous brain, in contrast to its homologs NAV1 and NAV2 (Figure 1A). Specifically, NAV3 expression is significantly lower in high‐grade gliomas (grade 4 astrocytoma and glioblastoma) compared to lower‐grade tumors (oligodendroglioma and grade 2 or 3 astrocytoma) (Figures 1B and S1A). Furthermore, NAV3 displays intratumoral heterogeneity—cells within the cellular tumor and pseudopalisading areas express less NAV3 compared to cells of the microvascular proliferation region or the infiltrating tumor (Figure 1C).

Although the downregulation of NAV3 in areas within the cellular tumor and its low level of expression in high‐grade GBM suggest that it acts as a tumor suppressor, survival data for newly diagnosed IDHwt GBM cases indicate that high NAV3 expression is associated with worse survival (Figure 1D). Interestingly, analysis of NAV3 expression at single‐cell level, based on data from Darmanis et al. [23], implies that the decreased levels of NAV3 within the tumor areas are due to a low percentage of neoplastic cells expressing NAV3 (Figure 1E, left), rather than lower expression levels compared to other cell types (Figure 1E, right). This perplexing expression pattern of NAV3 prompted us to further investigate its role in GBM cells.

### 2.2 NAV3 is more abundantly expressed in cells at the invading edge compared to the tumor sphere core

**FIGURE 2:**
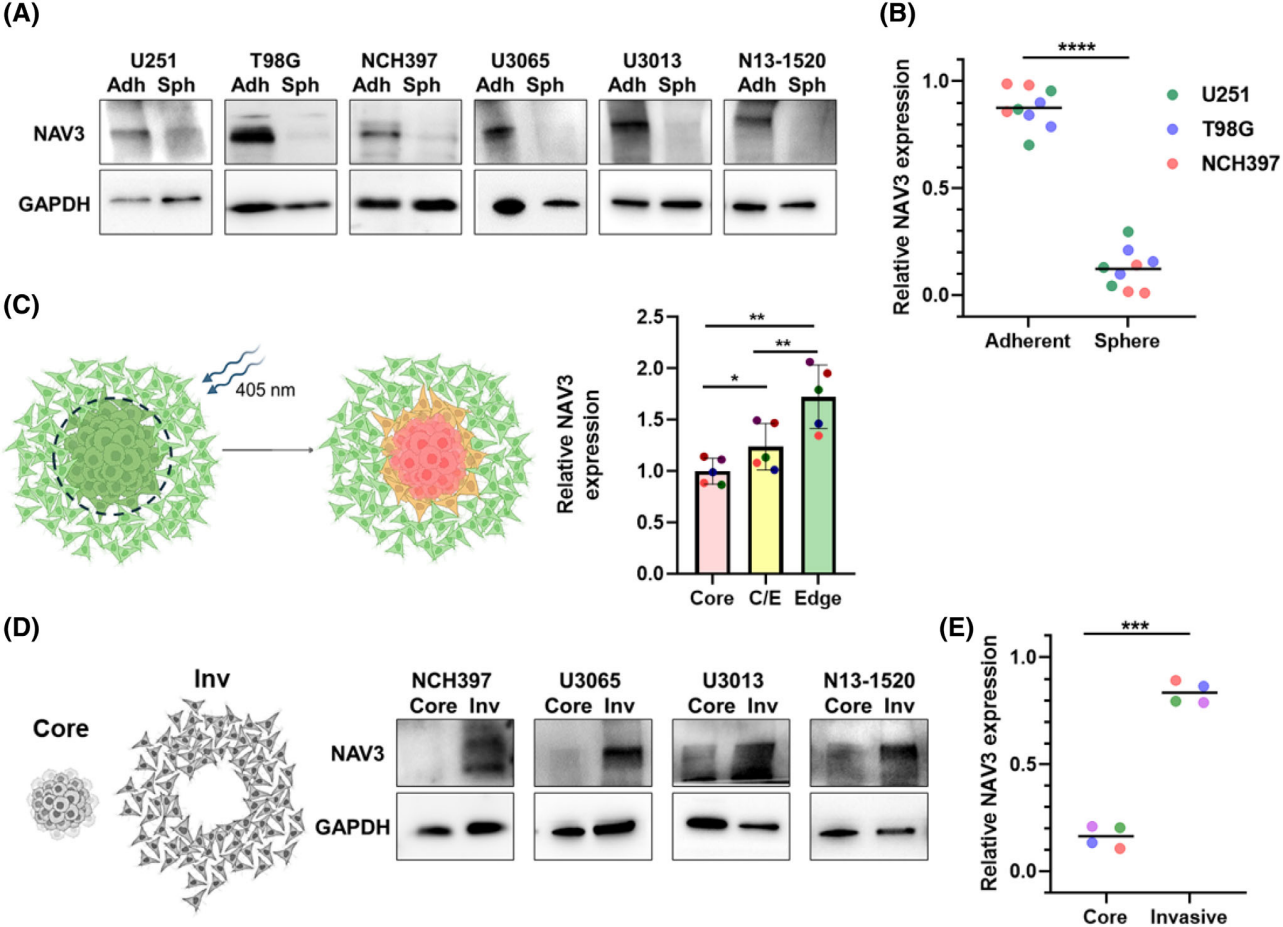
Differential expression of NAV3 in adherent and sphere‐cultured cells. (A) Two GBM cell lines and four patient‐derived GSCs were cultured either as adherent cultures (Adh) or in the form of tumor spheres (Sph) and probed for NAV3 expression using immunoblotting. Representative results are shown. (B) Quantification of NAV3 expression detected by western blot was conducted based on three independent replicates of each U251, T98G, and NCH397 cell lines. Statistical significance was determined using unpaired *t*‐test. (C) Schematic image of Dendra2 photoconversion strategy of tumor sphere core/edge cells (left) and quantification of NAV3 expression determined by flow cytometry analysis after photoconversion (right). Statistical significance was determined using repeated measures of one‐way ANOVA with post‐hoc Tukey test based on results from five independent experiments. (D) Tumor spheres prepared from four patient‐derived GSC cultures were plated and allowed to migrate. Subsequently, samples from sphere cores (Core) manually separated from invaded cells (Inv) were probed for NAV3 expression using immunoblotting. (E) Quantification of NAV3 expression detected by western blot shown as in (D). Statistical significance was determined using paired *t*‐test. (B), (C), (E): *p ≤ 0.05, **p ≤ 0.01, ***p ≤ 0.001, ****p ≤ 0.0001). Schematic illustrations in (C) and (D) were created using BioRender.com.

The lowest expression of NAV3 is found in pseudopalisading cells, which are situated within the cellular tumor mass in proximity of necrotic areas [24, 25]. The cellular milieu in these areas is characterized by high cell density and negligible access to substrate adhesion sites. Conversely, cells located at the infiltrating edge are dispersed within adjacent healthy tissue and interact with the surrounding ECM [26, 27]. To mimic these conditions experimentally, we generated paired samples of glioma cells cultured as tumor spheres under non‐adhesive conditions, and cells grown as adherent cultures from U251 and T98G GBM cell lines, as well as from four patient‐derived GBM stem‐like cell (GSC) cultures (NCH397, U3065, U3013, and N13‐1520). Across all tested samples, we observed decreased NAV3 expression in non‐adhesive, spheroid‐cultured cells compared to their adherently grown counterparts (Figure 2A, B).

To investigate whether spheroid‐cultured cells re‐express NAV3 upon migration from the sphere, reflecting cells disseminating from the tumor mass, we prepared U251 cells stably expressing the photoconvertible fluorophore Dendra2 (U251‐Dendra2). Illumination of this protein with blue light induces a spectral change [28], which is very stable in our cells for at least several hours. Selective photoconversion of cells of the inner spheroid allowed us to differentiate them during subsequent flow cytometry measurements.

Tumor spheroids derived from U251‐Dendra2 cells were allowed to adhere and spread for 48 h prior to photoconversion of the spheroid's inner region, easily distinguishable from the morphologically distinct, invasive cells (Figure 2C). Immediately post‐photoconversion, the samples were fixed and processed for flow cytometry analysis of NAV3 expression. This method allowed us to discern three distinct populations: (a) cells from the spheroid core (photoconverted, non‐adherent), (b) cells beneath the spheroid core and in its immediate vicinity (interface of core and migrated cells, photoconverted but adherent), and (c) adherent, invasive cells that migrated from the sphere (non‐photoconverted). Repeated experiments consistently showed a significant increase of NAV3 expression in adherent cells, with the invasive population (c) exhibiting the highest NAV3 levels (Figure 2C).

We confirmed this result using patient‐derived GSCs. Since the generation of Dendra2‐bearing variants was not feasible, we manually dissected the spheroid core from the adherent, out‐migrated cells. Across all tested cell cultures, we consistently observed an upregulation of NAV3 in cells that had migrated from the spheroid core (Figure 2D, E).

### 2.3 Overexpression of NAV3 promotes invasive behavior of GBM cells

**FIGURE 3:**
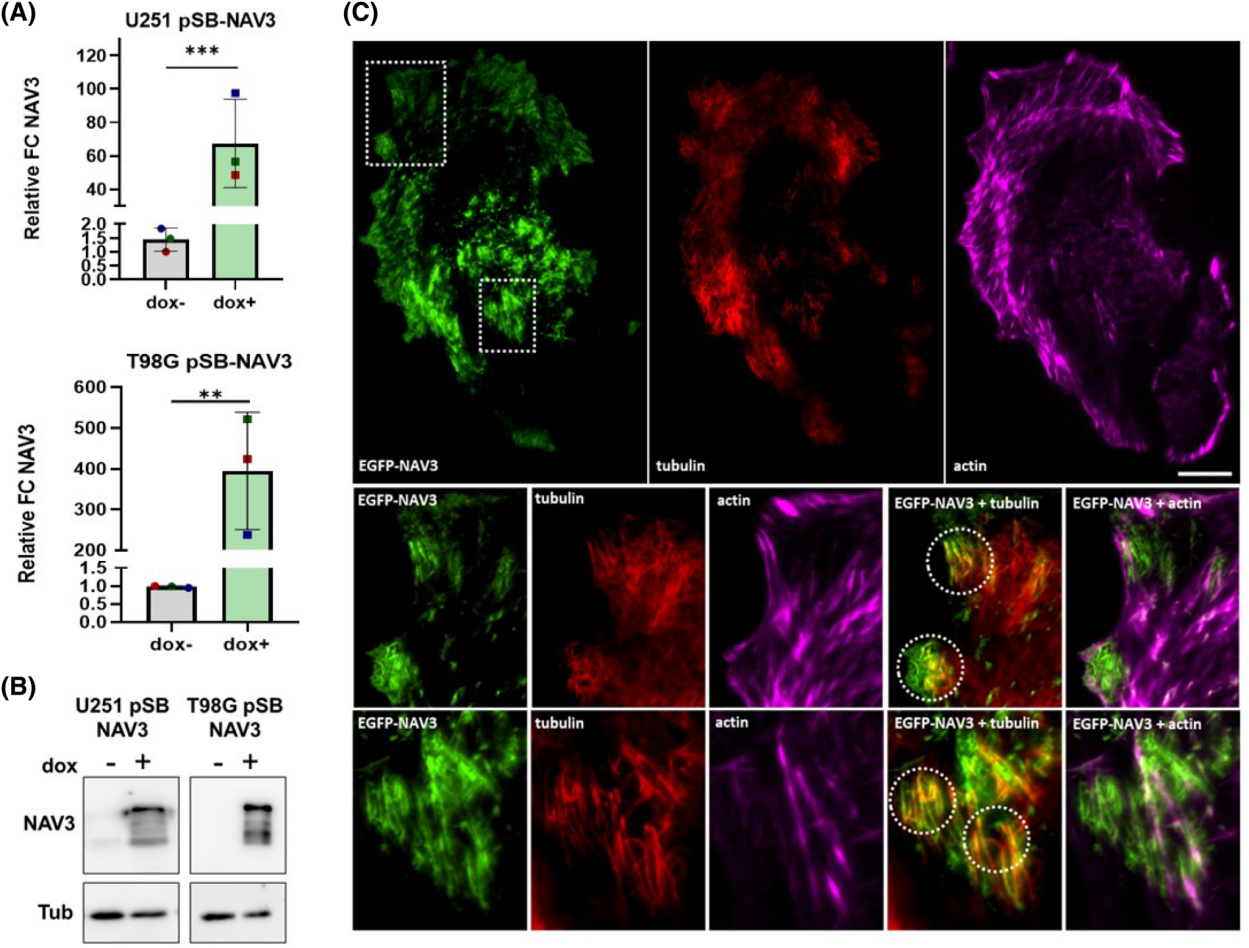
Inducible NAV3 overexpression in U251 and T98G GBM cell lines. U251 and T98G cells were transduced with Sleeping‐Beauty transposon‐based vector (pSB) carrying EGFP‐NAV3 under doxycycline (dox) inducible promoter. RT‐qPCR (A) and western blot (B) validation of NAV3 overexpression following dox treatment in U251 and T98G pSB‐NAV3 cell lines (FC‐fold change). Statistical significance was determined using ratio‐paired *t*‐test based on results from three independent experiments. **p ≤ 0.01, ***p ≤ 0.001. (C) Representative image of U251 pSB‐NAV3 dox + cell with immunofluorescently labeled Actin (magenta) and microtubules (red) visualized by TIRF microscopy depicting EGFP‐NAV3 localization. Image insets show colocalization of NAV3 with actin and tubulin (circled areas). Scale bar 20 μm.

We then assessed whether overexpression of NAV3 could directly modulate cell behavior. We transduced two GBM cell lines (U251 and T98G) with a transposon‐based vector harboring EGFP‐NAV3 under a doxycycline‐inducible promoter, thus establishing an inducible NAV3 overexpression (NAV3 OE) model system (referred to as U251 pSB‐NAV3 and T98G pSB‐NAV3; see Methods for more details). The overexpression of EGFP‐NAV3 was validated at both protein and mRNA expression levels (Figure 3A, B). In agreement with previously published results, we detected EGFP‐NAV3 colocalization with MT plus ends in both U251‐ and T98G pSB‐NAV3 cells (Figures 3C, and S2A, respectively). We also detected EGFP‐NAV3 signal together with actin filaments at the cell periphery, as previously described for the NAV1 [15, 16].

**FIGURE 4:**
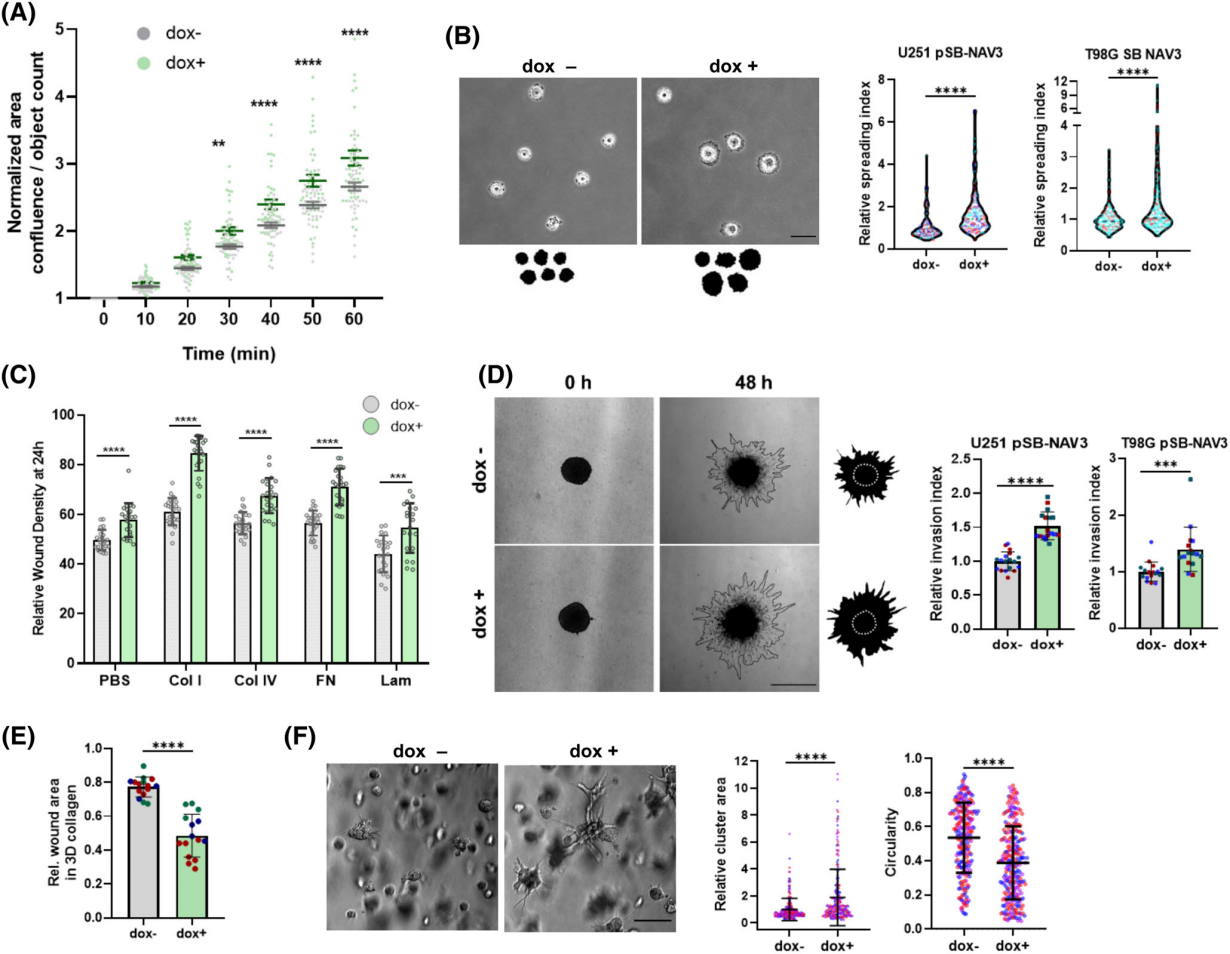
Overexpression of NAV3 promotes cell invasion‐associated behavior. U251 and T98G pSB‐NAV3 cell lines were subjected to cell adhesion, migration, and invasion assays. (A) Cell spreading dynamics of U251 pSB‐NAV3 cells ± dox on collagen I coated surface during the first hour after cell seeding. (B) Analysis of cell area during cell spreading. Representative images of U251 pSB‐NAV3 cells ± dox at 50 min after cell seeding (left) and quantification of relative cell area of both U251‐ and T98G‐pSB NAV3 cells ± dox (right), *n* >150 per condition. Scale bar 50 μm. (C) Results of 2D wound healing assay conducted using U251 pSB‐NAV3 cells ± dox on various ECM substrates, PBS as non‐ECM control. (D) Analysis of 3D collagen I spheroid invasion. Representative images of U251 pSB‐NAV3 spheroids ± dox embedded in 3D collagen before (0 h) and after invasion (48 h) (left) and quantification of relative cell invasion of both U251‐ and T98G‐pSB NAV3 cells ± dox (right), *n* >5 spheroids per biological replicate; scale bar 100 μm. (E) Results of 3D collagen I wound healing assay with U251 pSB‐NAV3 cells ± dox showing relative wound area after 20 h of wound closure. (F) Analysis of 3D matrigel invasion. Representative images of U251 pSB‐NAV3 cells ± dox embedded in 3D matrigel for 3 days (left) and corresponding quantification of cell cluster area and circularity ± dox (right), *n* >200, scale bar 100 μm. Data are shown as mean ± SD (A), (F) or as min‐max (C)–(E). Statistical significance was determined using one‐way ANOVA (A) and un‐paired *t*‐tests (B)–(F) based on results from at least three independent replicates (color‐coded in graphs); **p ≤ 0.01, ***p ≤ 0.001, ****p ≤ 0.0001.

Next, we assessed NAV3 OE cells for changes in motility and invasion‐associated behavior. To ensure that any observations were not influenced by differences in cell counts, we verified that NAV3 OE did not affect cell viability and proliferation (Figure S2B). Subsequently, since NAV1 was shown to crosslink actin and MTs, contributing to the extension of the cells' leading edge [16, 29], we measured the effect of NAV3 OE on cell spreading. Time‐course analysis of U251 pSB‐NAV3 ± dox cells spreading on collagen I‐coated surfaces showed an accelerated spreading of NAV3 OE cells compared to control cells (Figure 4A). Similarly, quantification of cell area 50 min after seeding demonstrated that NAV3 OE U251 and T98G cells were significantly larger than control cells (Figure 4B). We then used a wound healing assay with U251 pSB‐NAV3 ± dox cells on collagen I, collagen IV, fibronectin, and laminin‐coated substrates (Figure 4C), alongside uncoated plastic (Figure S2C) to measure cell migration. The results showed an increased migration speed of NAV3 OE cells in all conditions. NAV3 OE did not significantly affect cell adhesion to collagen I, collagen IV, and fibronectin (Figure S2D) and did not modify the expression of cell‐adhesion‐associated proteins (Figure S2E), indicating that NAV3 OE‐induced changes in cell spreading and migration were not caused by alternations of cell adhesion.

Following this, we measured the 3D‐invasion of U251 and T98G pSB‐NAV3 ± dox spheroids embedded in collagen I. This revealed a significant increase in invasion of NAV3 OE cells compared to controls (Figure 4D). The increased invasion rates of U251 NAV3 OE cells in collagen were also confirmed using a 3D wound healing assay (Figure 4E). Finally, U251 pSB‐NAV3 ± dox cells were cultivated in matrigel. Unlike control cells that grew mostly in compact cell aggregates, cells with NAV3 OE formed expansive clusters of cells with elongated cell protrusions (Figure 4F).

Collectively, our findings indicate that overexpression of NAV3 fosters invasive GBM behavior and suggest that the pro‐invasive phenotype seen in NAV3 OE cells is driven by modulation of cytoskeletal dynamics rather than changes in adhesion.

### 2.4 Low and high NAV3 levels associate with amoeboid and mesenchymal modes of invasion

**FIGURE 5:**
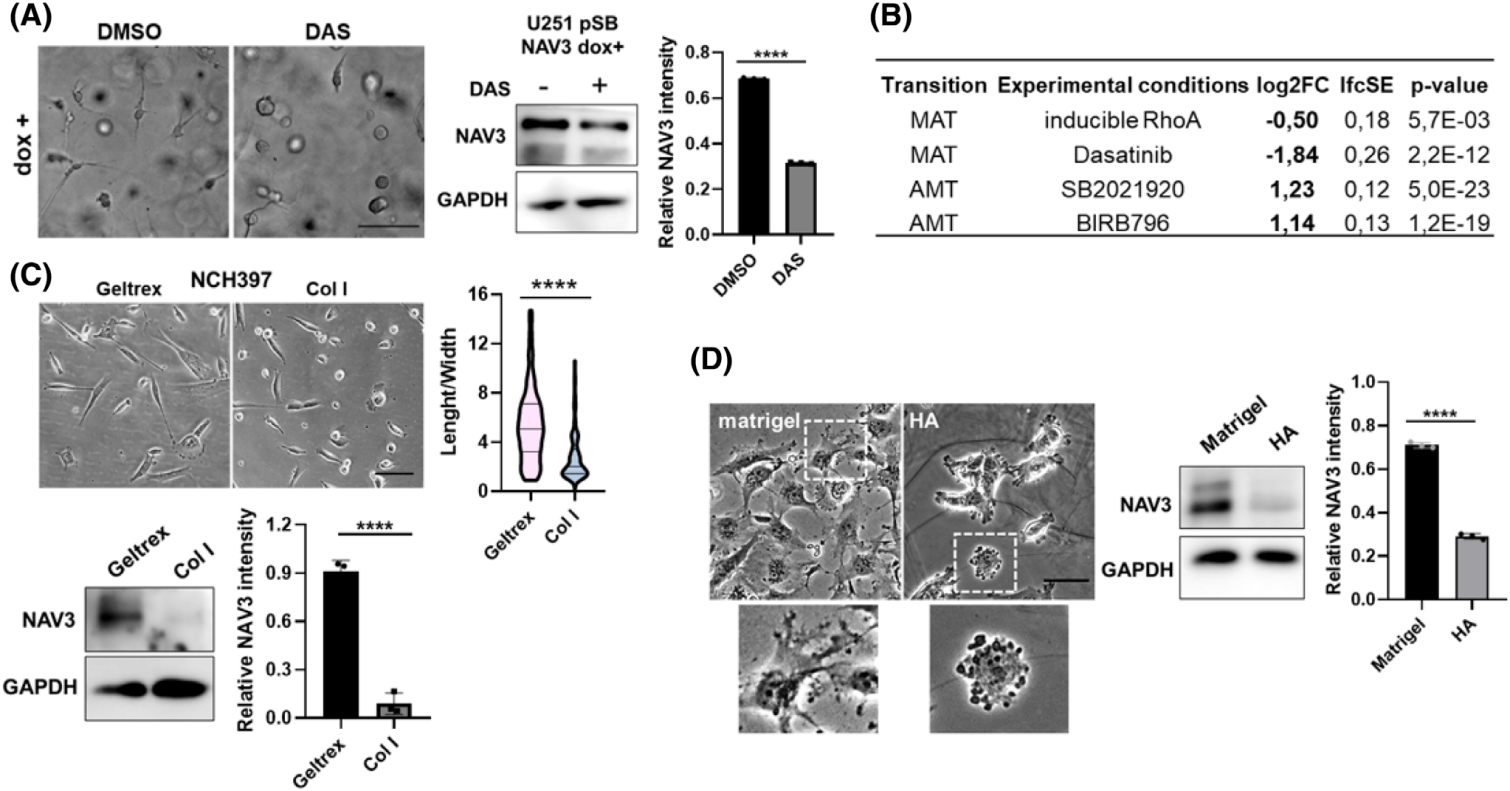
Differential NAV3 expression is associated with invasion plasticity. Cells with mesenchymal and amoeboid characteristics show high and low NAV3 expression, respectively. (A) Representative image of U251 pSB‐NAV3 dox + treated with DMSO or DAS in 3D collagen, scale bar 100 μm (right) and corresponding western blot detection of NAV3 (middle) with quantification of NAV3 expression based on three independent replicates (right). (B) Analysis of RNA‐seq data of cells undergoing invasion transitions shows decreased NAV3 expression in HT1080 fibrosarcoma cells after mesenchymal‐amoeboid transition (MAT) and an increase in NAV3 expression in A375m2 melanoma cells after AMT. See also Figure S3C. Representative image of patient‐derived NCH397 cells cultivated on either Geltrex or collagen I coated surfaces (left) and quantification of cell morphology on either substrate measured as length/width ratio (right). Scale bar 100 μm. Statistical significance of three independent replicates was determined using *t*‐test, *n* >140 per condition. Below, western blot detection of NAV3 corresponding to upper panel and quantification of NAV3 expression based on three independent replicates. (D) Representative image depicting U251 morphology on either matrigel or hyaluronic acid rich hydrogel (HA), inset shows close up of cells with mesenchymal‐like protrusions and amoeboid‐associated membrane blebs, respectively. Scale bar 100 μm (left). Western blot detection of NAV3 in U251 cells cultivated either on matrigel or HA (middle) and corresponding quantification of NAV3 expression based on three independent replicates (right). Statistical significance was determined using *t*‐tests, ****p ≤ 0.0001.

Phenotype plasticity stands as a prominent factor contributing to the frequent recurrence observed in GBM, with invasion plasticity posing a significant challenge in the pursuit of effective anti‐invasive therapies [30]. We thus investigated GBM cell morphology during invasion in association with NAV3 expression levels. U251 pSB‐NAV3 cells overexpressing NAV3 were treated with an Src kinase inhibitor, dasatinib, to induce the mesenchymal to amoeboid transition (MAT) [31]. Treated cells exhibited a transition to a round, amoeboid‐like phenotype, concomitant with a decrease in NAV3 expression (Figure 5A). Similarly, a reduction in NAV3 expression was observed following treatment of U251 cells with the focal adhesion kinase inhibitor PF‐562271, which also induced a shift from an elongated, mesenchymal‐like invasion phenotype to round, blebbing cells reminiscent of amoeboid invasion (Figure S3 A, B).

Next, we investigated changes in NAV3 expression levels during cancer invasion plasticity by analyzing our previously acquired transcriptomic data from fibrosarcoma and melanoma cells undergoing MAT [31] and amoeboid‐mesenchymal transition (AMT) [32] in 3D collagen. Intriguingly, this highlighted NAV3 as being upregulated in mesenchymally invading cells and downregulated in cells employing amoeboid invasion strategies in fibrosarcoma and melanoma cells (Figure 5B, Figure S3C), in line with results on GBM cells. Furthermore, the correlation between cell invasion phenotype and NAV3 expression levels was also established using a set of three melanoma cell lines: mesenchymal BLM, mixed WM3629, and amoeboid A375m2. High protein and gene expression levels of NAV3 were indicative of the mesenchymal invasion phenotype, whereas low NAV3 levels were detected in amoeboid cells (Figure S3D, E).

Individual ECM substrates exert distinct effects on GBM cell morphology and invasive behavior [33, 34]. Thus, we seeded NCH397 GSCs onto surfaces coated with either Geltrex or collagen I. Our observations revealed a marked alteration in cell morphology, characterized by a visible reduction in cell elongation and an increased number of rounded cells on collagen I, and this morphological transformation corresponded with a decrease in NAV3 expression (Figure 5C). Notably, we noted a similar phenotypic plasticity with a transition to rounded cells on collagen, accompanied by a decrease in NAV3 expression, in N13‐1520 GSCs, but not in U3013 or U3065 GSCs. These findings highlight heterogeneity in invasion phenotype plasticity across individual GBM cultures (Figure S3F).

We also assessed the changes in NAV3 expression using U251 cells cultured either on matrigel, abundant in basement membrane proteins resembling attachment to blood vessels, or on hyaluronic acid (HA)‐rich hydrogel, to mimic native brain ECM. When seeded on matrigel, U251 cells exhibited an elongated phenotype with numerous protrusions. Conversely, they adopted a rounder morphology with evident membrane blebbing on HA hydrogels (Figure 5D), consistent with the previously described pro‐amoeboid role of HA‐rich ECM [13, 35]. Consistently, this transition was accompanied by a downregulation of NAV3 expression on HA‐based substrates compared to matrigel (Figure 5D).

Taken together, our results show that the nature of the ECM affects NAV3 expression in GBM cells, which changes their invasive phenotype. Based on results from GBM cell lines and GSCs, but also fibrosarcoma and melanoma cells, we demonstrate that low NAV3 expression is associated with the round, amoeboid invasion phenotype, whereas high NAV3 levels correlate with the elongated morphology and protrusion‐dependent mesenchymal invasion phenotype.

### 2.5 Analysis of NAV3 expression in mouse glioblastoma xenografts

**FIGURE 6:**
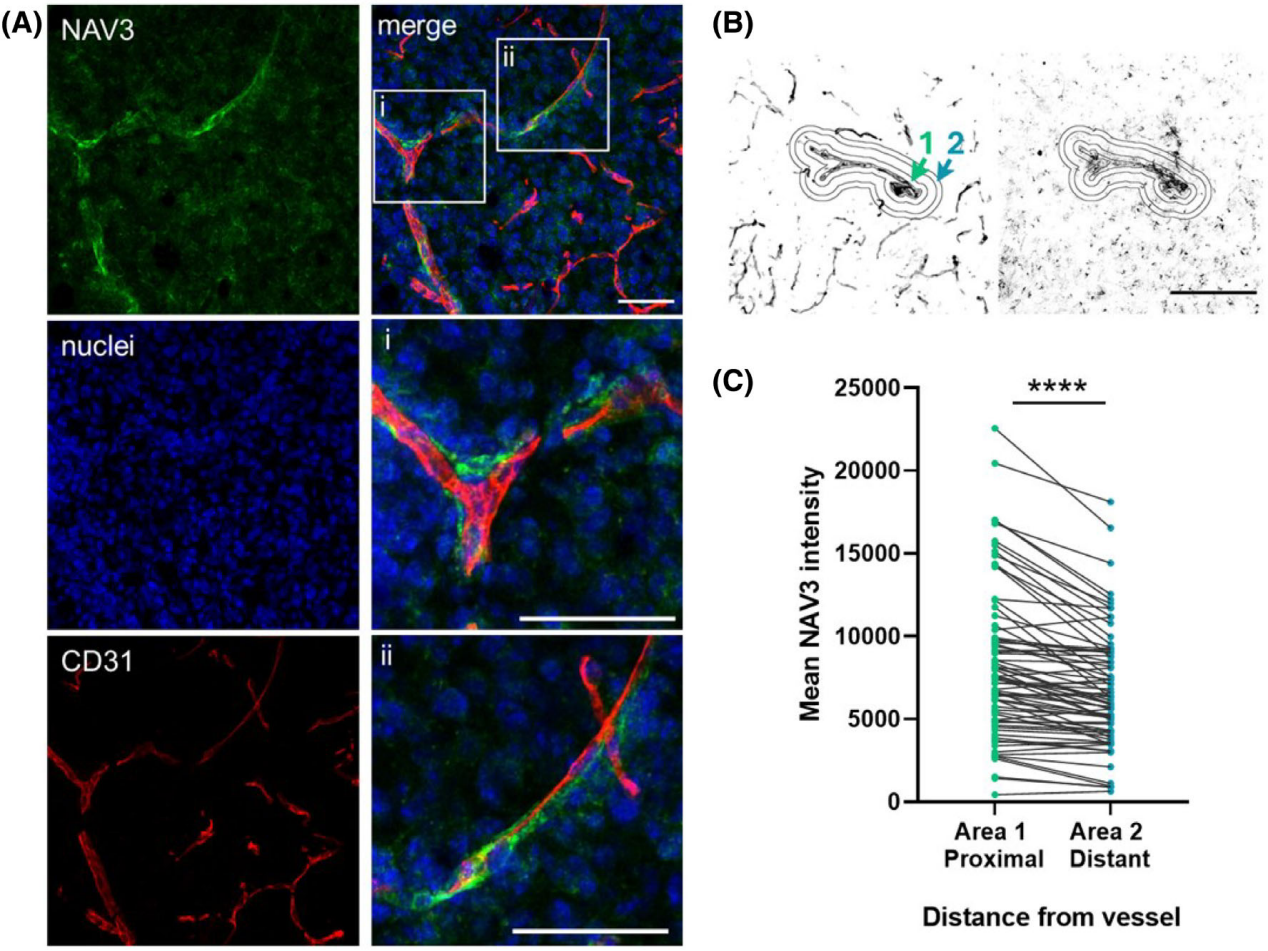
Immunohistochemical detection of NAV3 in glioblastoma xenografts in mice. (A) Representative image of immunohistochemical detection of NAV3 (green) and the marker of endothelial cells CD31 (red) in glioblastoma xenografts from NCH397AG GSC culture. Left, individual channels right merged channels. Scale bar 50 μm. (B) Schematic image depicting the quantification strategy for NAV3 intensity in areas proximal or distal to vessels. Left image CD31 signal, right image NAV3 signal. Scale bar 50 μm. (C) Results of quantification of NAV3 intensity based on staining of blood vessels (*n* = 9) were determined using paired *t*‐test, ****p ≤ 0.0001.

To corroborate our in vitro findings, we examined NAV3 expression in mouse GBM xenografts generated by implanting NCH397 GSCs, cultured both as an adherent culture (NCH397AG cells) and as non‐adhesive spheres (NCH397A cells), into the brain of immunodeficient mice. Positive NAV3 staining was observed in xenografts derived from NCH397AG cells (Figure 6A), as well as non‐adhesive sphere cultured NCH397A cells, which do not express NAV3 in vitro (Figure 2A), showing that NAV3 can be re‐expressed in the brain microenvironment. Moreover, in both cases, the intensity of NAV3 expression was significantly higher in areas adjacent to blood vessels compared to regions distal from blood vessels (Figure 6B, C). This observation corroborates our in vitro findings, demonstrating that cell interaction with basement membrane‐like matrix promotes NAV3 expression. Altogether, our results point to NAV3 as a key molecule overexpressed following GBM cell contact with the basement membrane matrix and essential for the acquisition of a mesenchymal invasion phenotype.

## 3 DISCUSSION

GBM exhibit decreased NAV3 expression compared to low‐grade gliomas, and this has led to the perception of NAV3 as a negative regulator of GBM progression. Similarly, NAV3 was previously proposed as a suppressor of migration and metastasis in colon and breast cancer [36, 37]. However, our findings show that NAV3 is re‐expressed in migrating cells and its overexpression in fact promotes mesenchymal invasive behavior, which challenges its conventional classification as a tumor suppressor.

For NAV3 OE studies in glioma cell lines, we utilized a doxycycline‐inducible system to allow for direct comparison of cells with or without induced overexpression of NAV3. Overexpression of NAV3 was well tolerated in cells and did not cause any abnormal phenotypes, nor were the cells affected in terms of viability or morphology. Although we cannot fully exclude that induction of higher than physiological levels of NAV3 in our GBM cell lines could to some extent influence their behavior, we consistently demonstrate that NAV3 OE promotes invasive behavior of GBM cells (Figure 4). Notably, in line with our results, an invasive subline of U87 GBM cells showed higher NAV3 expression compared to parental cells [38] (Figure S1B).

Furthermore, NAV3 expression demonstrates spatial heterogeneity within distinct histologically defined regions of GBM tumors [39] with highest NAV3 levels detected at the leading edge, although large presence of non‐malignant cells in this area may contribute to this observation [40]. Nevertheless, single‐cell RNA‐seq data of GBM disclosed that a small fraction of GBM cells express NAV3 at levels comparable to neuronal tissue (Figure 1E) [23]. Interestingly, a recent study on melanoma cells described a similar trend. NAV3 was found to be decreased in thicker, more progressed melanoma, despite being identified at the leading edge in melanoma cells and shown to contribute to protrusive activity of cells in 3D collagen [41].

We posit that this puzzling NAV3 expression pattern reflects the phenotypic heterogeneity of cancer cells. For instance, GBM cells, especially GSCs, display growth pattern variability and can be grown as adherent or sphere‐forming cultures in vitro. Those cell lines that adopted an attached, elongated morphology had increased NAV3 expression levels compared to those that maintained a sphere growth pattern [42], (Figure S1 C, D). A similar trend of differential NAV3 expression, with low levels observed in cells residing within the tumor sphere core and higher levels in cells at the tumor spheroid edges, respectively, was also detected in our samples from both established GBM cell lines and patient‐derived GSC cultures. Consistently, a previous study identified NAV3 as one of the top upregulated genes in GBM cells that have migrated out from spheroids compared to cells remaining in the undispersed core of the spheroid [43]. The phenotypic differences between tumor core and tumor edge cells are controlled by both local environmental factors and inherent cellular characteristics. These intrinsic cellular attributes may persist within the cells even after their isolation from the initial microenvironment [44, 45]. Although the cells located at the infiltrating edge may be perceived as more invasive, tumor core cells also significantly contribute to GBM invasive behavior by transmitting pro‐invasive signaling to cells at the infiltrative margin or mediating increased therapy resistance [44, 46].

Our results point to a connection between NAV3 and invasion plasticity, with round, amoeboid cells displaying lower levels of NAV3 compared to mesenchymally invading, elongated cells. Importantly, this association appears to be conserved across various cancer types, including GBM, fibrosarcoma, and melanoma. Interestingly, previous studies showed that GBM cells derived from the tumor core and invasive margin had distinct morphologies, with core cells being rounder compared to the more elongated cells isolated from the tumor edges [44, 47], which would be indicative of the low and high NAV3 levels we detected in our spheroid core/edge samples and consistent with NAV3 levels detected in amoeboid/mesenchymal cells. NAV3 can contribute to the morphological shifts through its modulation of MT dynamics; however, the intricate mechanisms governing its diverse expression patterns remain to be elucidated.

The nature of the ECM can largely dictate the choice of the invasion phenotype with stiff, rigid ECM preferring mesenchymal traits and soft, porous ECM promoting characteristics typical of amoeboid cells [48, 49], which holds true for GBM cells as well [10, 50, 51]. Hence, this stresses the necessity to select an appropriate 3D environment and consider its constraints during experimental design. Collagen expression is elevated in GBM [52] and enables the manifestation of both amoeboid and mesenchymal invasion phenotypes of GBM cells [53], rendering it an appropriate 3D matrix for GBM invasion studies. Notably, cells cultured in patient‐derived decellularized matrix demonstrated a similar representation of round amoeboid cells and elongated mesenchymal cells, indicating a substantial contribution of amoeboid invasion mode to GBM infiltration [53]. Our results demonstrated that GSCs can adapt to alterations in the ECM. Specifically, GSCs can transition between elongated, protrusion‐rich morphologies indicative of mesenchymal invasion, and rounded, bleb‐containing morphologies characteristic of amoeboid invasion, as the ECM shifts from basement membrane‐like Geltrex to collagen I. Notably, these ECM‐induced morphological alterations correlate with concomitant high and low levels of NAV3, respectively.

Using GSC xenografts in murine models, we were able to identify NAV3 positive cells within the GBM tumors and demonstrate that cells migrating along vessels exhibit increased NAV3 expression levels compared to those located more distally from the vasculature. This augmented NAV3 expression might be ascribed to the interactions of GBM cells with constituents of the vascular basement membrane. This is consistent with our observations indicating elevated NAV3 levels in cells cultured on matrigel compared to those on hyaluronic acid. Furthermore, the spatial directionality provided by vascular structures may independently contribute to NAV3 upregulation, as evidenced by a previous study demonstrating nearly two‐fold increased NAV3 expression in GBM cells cultured on aligned fibers [54]. Notably, NAV3 was identified to be upregulated in GBM cells cocultured in 3D hydrogels along with perivascular cells [55], which further underlines the role of microenvironmental interactions in modulating NAV3 expression. Altogether, these results insinuate that NAV3 is associated with perivascular migration of GBM cells allowing directed invasion, although the mechanism of its action remains to be elucidated.

Collectively, our results imply that the decrease of NAV3 expression levels in GBM likely stems from a large tumor core containing necrotic regions, exhibiting low NAV3, rather than being indicative of a tumor suppressor role of NAV3 in glioma cells. Additionally, the variance in NAV3 levels may be attributed to a shift in the invasion strategy from protrusion‐dependent mesenchymally invading cells, which are commonly identified migrating along blood vessels and characterized by high NAV3 expression, to individually dispersed cells within the brain parenchyma, exhibiting more amoeboid features and diminished NAV3 expression.

## 4 CONCLUSION

NAV3 expression is contextually regulated and responsive to the surrounding microenvironment along with associated alterations in cell morphology and invasive behavior. Moreover, NAV3 levels reflect and participate in creating intratumoral heterogeneity, with high NAV3 marking areas of GBM perivascular infiltration. Understanding the molecular mechanisms of this diversity within GBM is essential for refining therapeutic strategies targeting its invasiveness. Our data contribute to the understanding of GBM invasion and its plasticity, highlighting the association of NAV3 with these traits.

## 5 MATERIALS AND METHODS

### 5.1 Data analysis

NAV3 expression data in adult diffuse gliomas were downloaded from the Gliovis data portal (http://gliovis.bioinfo.cnio.es/) [22]. Only primary tumors were included in the analysis. For the TCGA‐GBMLGG dataset, the tumors were reclassified based on the phenotypic information available in the TCGA dataset according to the current WHO 2021 classification into the following categories: (1) Glioblastoma, IDHwt (glioblastoma histology and/or chromosome 7 gain/chromosome 10 loss and/or TERT promoter mutation), (2) Oligodendroglioma (IDHmut, 1p19q co‐deleted, grade 2 or 3), (3) Astrocytoma, IDHmut (grade 2 or 3), and (4) Astrocytoma, IDHmut, grade 4. Single‐cell RNA‐seq data were downloaded from http://gbmseq.org on November 16, 2021. Further descriptions and source data are available in the Supplementary Tables. Statistical analysis and data visualization were performed using GraphPad Prism.

### 5.2 Cell culture

Cells were cultured at 37°C with 5% CO_2_, routinely passaged and checked for contamination. Commercial U251 and T98G cell lines were cultivated in DMEM (Sigma) with 4.5 g/L L‐glucose, L‐glutamine, and pyruvate, supplemented with 10% fetal bovine serum (Sigma) and 50 μg/mL gentamicin (Sigma). Glioma stem‐like cells NCH397 were derived by authors of this study as described previously [56] (see Supplementary Methods for more detail). Patient‐derived glioblastoma cells U3013 and U3065 were acquired from the Human Glioblastoma Cell Culture resource (www.hgcc.se) at the Department of Immunology, Genetics and Pathology, Uppsala University, Uppsala, Sweden, for more information see Reference [57]. N13‐1520 cells were obtained from GlioTex (Institut du Cerveau et de la Moelle Epinière, ICM, F‐75013, Paris, France) [58], all institutions having the necessary ethical agreements to collect GBM samples from informed patients. N13‐1510, U3065, and U3013 cells were cultivated in serum‐free DMEM‐F12 (Sigma) mixed 1:1 with Neurobasal‐A Medium (Sigma), and NCH397 cells in DMEM‐F12 minus phenol red (Sigma). For all GSCs, culture media was supplemented with 1% Glutamax (Gibco), 1% penicillin–streptomycin (Sigma), 2% B‐27™ Supplement (Gibco), and 20 ng/mL EGF and 20 ng/mL FGF (PeproTech). GSCs were propagated in non‐adherent cell culture flasks as spheres or on Geltrex (0.05 mg/mL; Thermo Fisher Scientific) coated flasks in case of adherent GSC cultures.

For the generation of U251 pSB‐NAV3, T98G pSB‐NAV3, and U251‐Dendra2 stable cell lines, cells were transfected with DNA constructs using polyethylenimine (Polysciences) and enriched for positive cells using cell sorting. For more information, see Supplementary Methods. For inducing the expression of EGFP‐NAV3 in U251 and T98G pSB‐NAV3 stable cell lines, 250 ng/mL doxycycline (dox; Sigma) was added to cell medium. Dasatinib (LC Laboratories) was used at 1 μM concentration, and DMSO (Sigma) served as a control.

### 5.3 Tumor spheroid cultures

Glioma tumor cell spheroids were prepared by culturing cells in a 3D Petri Dish® (Microtissues®; #12‐81) according to manufacturer's protocol. Briefly, micro‐molds were prepared using 2% agarose in 0.9% of NaCl in PBS and cells in suspension (2.1 × 10^3^ cells/μL) were added and left to settle before overlaying with culture media. Homogenously large spheroids were formed within 48–72 h. Next, the spheres were lyzed directly (for spheroid samples) or plated, and cells left to migrate for another 48 h (for the preparation of core vs. invasive samples), after which spheroid cores were manually separated by careful aspiration with a pipette tip.

### 5.4 Dendra2 photoconversion

U251 cells with stable expression of photoconvertible Dendra2 protein were grown to form spheroids (see above), which were then manually transferred to an 8‐well Ibidi dish (6–9 spheroids/well), left to attach, and overlaid with a culture medium. After 48 h, the medium was exchanged for phenol red‐free medium. The core of the spheroids was photoconverted using Nikon CSU‐W1 microscope (10× objective, 405 nm laser with 10% intensity, 100 ms dwell time). Photoconverted spheroids were detached with trypsin, and the spheroid cores separated by sedimentation and further disrupted by pipetting. Cells were fixed using 4% PFA (Sigma) followed by 90% ice‐cold methanol, labeled for NAV3 with 0.2 μg/100 μL NAV3 primary antibody (HPA032111; Sigma) in 0.5% BSA for 1 h at RT and 0.2 μg/100 μL AlexaFluor 405 nm secondary antibody (A‐31556; Thermo Fisher Scientific) in 0.5% BSA for 30 min at RT in the dark. The BD LSR Fortessa cytometer was used to measure the fluorescent intensity of labeled NAV3 after photoconversion, fixation, and immunolabeling.

### 5.5 Morphology experiments

For morphology experiments, GSCs were seeded on Geltrex (0.05 mg/mL; Thermo Fisher Scientific) or collagen I (0.05 mg/mL; Gibco) and imaged after 24 hs with the Olympus IX 70 microscope. For U251 morphology experiments, thin layers of matrigel (Corning® Matrigel® Basement Membrane Matrix; Sigma) or HyStem‐HP™ HA Based Hydrogel (Sigma) coated surfaces were prepared in 48‐well plates (50 μL of matrix per well) according to manufacturer's recommendations. Briefly, matrigel was thawed on ice and pipetted using pre‐chilled tips. HA Based Hydrogel was prepared following the protocol for standard stiffness (1%) by mixing the components Extralink, Glycosil, and Gelin‐S in 1:2:2 ratio, respectively. After gelation, 3 × 10^4^ cells per well were added. After 48 h, cells were imaged using Nikon ECLIPSE TE2000‐S microscope. For both GSCs and U251 cells, cell morphology was measured as length/width ratio manually using the Fiji ImageJ Software [59].

### 5.6 In vitro fluorescence microscopy

Cells were grown overnight on Ibidi plates (μ‐Slide 8 Well high Glass Bottom) in the presence of 250 ng/mL of doxycycline. One hour prior to observation, cells were treated with SPY555‐actin (SC202; Spirochrome), and SPY650‐tubulin (SC503; Spirochrome) using 1000× dilution. Images were acquired on Nikon Ti‐E H‐TIRF microscope equipped with Nikon CFI Apo TIRF 60× Oil, NA 1.49.

### 5.7 Immunoblotting

Protein lysates were prepared from either 2D or 3D cell cultures (as indicated in the text). For 2D protein lysates, glioma cells were harvested and transferred to 1× SDS lysis buffer (1% SDS, 10% glycerol, 60 mM Tris, pH 6.8) or in case of NCH397 GSC 1× RIPA buffer with protease inhibitors (Sigma). In case of 3D protein lysates, cells were cultivated for 30 h at a density of 200,000 cells per 500 μL of 3D collagen gel. Gels from two wells per sample were transferred to 2× SDS lysis buffer and homogenized using Tissue Tearor (BioSpec Products). Protein concentration of the samples was measured using the DCTM Protein Assay (Bio‐Rad Laboratories) and samples were adjusted to equivalent protein concentration. DTT (final concentration 50 mM) and bromophenol blue (final concentration 30 μM) were added and samples incubated at 95°C for 10 min. Samples were run on 6%–15% SDS‐polyacrylamide gradient gels and transferred onto nitrocellulose membrane using wet overnight blotting for effective transfer of proteins with high molecular weight (25 V overnight at 5°C, transfer buffer: 25 mM Tris and 192 mM glycine in 20% methanol). Membranes were blocked in TTBS buffer with 4% BSA and incubated with primary antibodies in TTBS with 2% BSA at 4°C overnight. The following primary antibodies were used: NAV3 (HPA032111; Sigma) and α‐Tubulin (2144S; Cell Signaling) at 1:1000 dilution and GAPDH (MA5‐15738; Thermo Fisher Scientific) at 1:5000 dilution. The Western blot images shown are representative of 3 independent biological replicates unless stated otherwise. Quantification of band intensities was performed using Image J; the intensities of specific proteins were normalized to the reference protein signal to adjust for protein loading.

### 5.8 RT‐qPCR

For RNA extraction, cells were washed quickly with PBS, and 1 mL RNA lysis solution was added (60% v/v water‐saturated phenol, 3.25 M guanidine thiocyanate, 400 mM sodium acetate buffer pH 4.0, 0.4% w/v N‐lauroylsarcosine and 160 mM 2‐mercaptoethanol in ddH_2_O). RNA was isolated using a modified Trizol method. Briefly, 200 μL of chloroform was added to each sample and vortexed. After 10 min incubation, samples were centrifuged (18,000× *g*, 4°C, 30 min). The upper phase was transferred to a fresh tube, and 600 μL of isopropanol was added to precipitate the RNA. After washing, RNA was diluted with RNase‐free water to a final concentration of 0.5 μg/μL and used for reverse transcription using oligo(dT) primers. RT‐qPCR was performed using 1× SYBR green mix, as described previously [49], using CFX384 Real‐Time PCR Instrument (Bio‐Rad). Cq values were exported, and relative expression was calculated using the 2^−ΔΔ*CT*^ method. Primers used were: NAV3 forward: 5′‐CACCGACACACTGATGCCAAGATT‐3′ and reverse: 5′‐AAACAATCTTGGCATCAGTGTGTC‐3′; reference gene GAPDH forward 5′‐GCATGGACTGTGGTCATGAG‐3′ and reverse 5′‐CTGCACCACCAACTGCTTAG‐3′.

### 5.9 Cell spreading

For analysis of cell spreading dynamics, 96‐well plates were coated overnight with 10 μg/mL collagen I (Millipore). Cells were grown ± dox 1 day prior to the experiment and starved in serum‐free media for 3 h before seeding 5000 cells per well. Plates were then transferred to the Incucyte S3 microscope (Sartorius) and images were acquired every 10 min for 2 h. Area confluence normalized to object count per image was calculated using the Incucyte Live‐Cell Analysis Systems. Data from three independent biological experiments were combined and average values statistically evaluated using one‐way ANOVA in GraphPad Prism. For measurements of cell area during spreading, cells pre‐treated ± dox were brought to suspension and replated. After 50 min of cell spreading, cells were imaged using Nikon ECLIPSE TE2000‐S microscope. Cell area was measured using Fiji ImageJ Software. Data from three independent biological experiments were statistically evaluated in GraphPad Prism using unpaired *t*‐tests.

### 5.10 Wound healing

A suspension of 40,000 cells in cell culture medium (± dox) was added to each well of a 96‐well plate (Sartorius, BA‐04855). The next day, a scratch was made using the Incucyte 96‐Well Woundmaker Tool (Sartorius, 4563), cells were washed twice and overlaid with cell culture medium (± dox). Subsequently, the plate was transferred to an Incucyte S3 microscope and images were acquired every 2 h for 24 h. Relative wound density was determined using Incucyte Scratch Wound Analysis Software (Sartorius, 9600‐0012). For wound healing on coated surfaces, the 96 well plates were coated with 50 μg/mL (100 μL per well) of collagen I (Millipore), collagen IV (Cultrex), fibronectin (Merck), or laminin (MP Biomedicals) diluted in PBS or PBS alone as a control for 3 h prior to seeding the cells. Average values of relative wound densities from three independent biological experiments were then calculated using GraphPad Prism, and statistical significances were determined by unpaired *t*‐tests.

### 5.11 3D migration and invasion assays

For 3D assays, collagen I matrix was prepared using buffer solution and rat tail collagen type I (in‐lab prepared). The resulting composition of the collagen gels was 1 mg/mL collagen, 1× RPMI medium, 15 mM HEPES, 1% fetal bovine serum, and 50 μg/mL gentamicin. Work was done on ice to prevent polymerization. For 3D migration, a modified wound healing assay was conducted. Briefly, 300 μL of 1 mg/mL collagen matrix was pipetted to a 24‐well dish on ice. After 30 min polymerization at 37°C, a culture insert with a defined cell‐free gap (Ibidi, 81,176) was placed on top of the collagen, and 100 μL of cell suspension (4 × 10^6^ cells/mL pre‐cultivated ± dox for 48 h) was transferred into the insert. Cells were left to attach for 4 h, after which inserts and excess medium were removed and cells overlaid with 500 μL collagen. After 30 min, 1 mL of cell‐free medium was added to each well. Images of the wounds were taken at 0 and 21 h using Leica DMi8 microscope. Migration was assessed as a decrease of cell‐free area in time quantified by Fiji ImageJ Software. For 3D spheroid invasion, spheroids prepared as described above (± dox) were transferred between two layers of the 3D collagen matrix in a 96‐well plate (1 spheroid per well) and overlaid with cultivation medium (± dox) after polymerization. Images of the spheroids were taken immediately after embedding into collagen (before) and after 48 h (after). The area of the spheroids before and after invasion was measured using Fiji ImageJ Software. For 3D invasion into matrigel, cells pre‐cultivated ± dox for 48 h were brought to suspension, carefully mixed 1:10 with matrigel (Corning® Matrigel® Basement Membrane Matrix; Sigma) and 100 μL of the matrix containing cells was added per well in 96‐well plates on ice. After gelation at 37°C, cell culture medium ± dox was added. The area and circularity of cell clusters were measured after 72 h using Fiji ImageJ Software. Data from three independent biological experiments were statistically evaluated in GraphPad Prism using unpaired *t*‐tests.

### 5.12 Generation of orthotopic xenografts

The experimental use of animals was approved by The Commission for Animal Welfare of the First Faculty of Medicine, Charles University in Prague, and the Ministry of Education, Youth and Sports of the Czech Republic according to animal protection laws. Glioma cells (500,000 cells/5 μL) were implanted in the right brain hemisphere of 6–8 weeks old NOD‐Rag mice (NOD.129S7(B6)‐Rag1tm1Mom/J; The Jackson Laboratory, Bar Harbor, ME, USA), as described previously [60]. Animals were euthanized under deep anesthesia after 15 weeks, with one exception of 11 weeks due to neurological symptoms appearing earlier. Afterwards, the brains were frozen and stored at −20°C.

### 5.13 Immunohistochemistry

Frozen sections (10 and 50 μm) were fixed with ice‐cold methanol (5 min at RT), permeabilized with 0.1% Triton‐X100, blocked with 5% bovine serum albumin (BSA) in 100 mM Tris/HCl—154 mM NaCl, pH 7.5 (TBS) and incubated 1 h at RT with primary NAV3 antibody (HPA032111; Sigma) 1:300 in TBS with 1% BSA, primary mouse CD31 (NB 100‐1642; Novusbio) 1:100 in TBS with 1% BSA. After washing, the slides were incubated with the corresponding Alexa Fluor 488‐ and 546‐conjugated secondary antibodies (Thermo Fisher Scientific, 1:500, 1 h at RT). Hoechst 33258 (50 ng/mL; Sigma) and ToPro (400 μM; Thermo Fisher Scientific) were used for nuclear counterstaining. The sections were then mounted in Aqua Polymount (Polyscience) and confocal images were taken using Stellaris 5 Confocal microscope (Leica).

### 5.14 Image quantification of IHC staining

Image analysis was performed in ImageJ. A stack was created from CD31 and NAV3 images, and individual vessels were outlined with the freehand selection tool. The tool “make a band” in ImageJ was used to select an area in close proximity (0–9 μm) and distant (25–34 μm) from the vessel and mean NAV3 signal intensity was calculated after background subtraction. Randomly selected blood vessels from several independent sections from four mice were analyzed (3 implanted with NCH397A cells, 1 with NCH397AG cells). Statistical significance was assessed in GraphPad Prism using paired *t‐*tests.

## AUTHOR CONTRIBUTIONS

All authors contributed to this work. *Conceptualization and study design*: AŠ, JB, DR, PB, and SEM. *Experimental investigation and acquisition of data*: AŠ, MP, OT, AL, RM, EGB. *Data visualization*: AŠ. *Study supervision, funding, and resources*: AŠ, JB, DR, PB, AŠe, SEM. *Writing—original draft*: AŠ. *Writing—review and editing*: AŠ, PB, and SEM. All authors read and approved the final manuscript.

## CONFLICT OF INTEREST STATEMENT

The authors declare no competing or financial interests.

## ETHICS STATEMENT

All experiments in this study were conducted in compliance with relevant guidelines and regulations. Glioma stem‐like cell cultures were derived by the respective institutions following written consent from patients. The experimental use of animals was approved by The Commission for Animal Welfare of the First Faculty of Medicine, Charles University in Prague, and the Ministry of Education, Youth and Sports of the Czech Republic according to animal protection laws.

## Supporting information


**Data S1:** Supporting Information

**Data S2:** Supporting Information

## Data Availability

We confirm that the primary data of this manuscript will be made available for sharing when requested.
