# Supplementary material for: Microtubule‐associated NAV3 regulates invasive phenotypes in glioblastoma cells
Source: Brain Pathol. 2024 Aug 3;35(1):e13294. doi: 10.1111/bpa.13294 (PMC11669409; doi:10.1111/bpa.13294)
Supplement: Supplementary file 1 — Data S1: Supporting Information [file BPA-35-e13294-s001.docx]

*Supplementary file for*

**Microtubule-associated NAV3 regulates invasive phenotypes in glioblastoma cells**

Aneta Škarková#^1^, Markéta Pelantová^1^, Ondřej Tolde^1^, Anna Legátová^1^, Rosana Mateu^2^, Petr Bušek^2^, Elena Garcia-Borja^2^, Aleksi Šedo^2^, Sandrine Etienne-Manneville^3^, Daniel Rösel^1^ and Jan Brábek#^1^

^1^ Laboratory of Cancer Cell Invasion, Department of Cell Biology, BIOCEV, Faculty of Science, Charles University, Vestec, Czech Republic.

^2^ Laboratory of Cancer Cell Biology, Institute of Biochemistry and Experimental Oncology, First Faculty of Medicine, Charles University, U Nemocnice 478/5, Prague 2, 128 53, Czech Republic

^3^ Cell Polarity, Migration and Cancer Unit, Université Paris Cité, UMR3691 CNRS, Institut Pasteur, Paris, France

# Corresponding author

**Key words :** glioblastoma, NAV3, invasion, amoeboid, mesenchymal

**SUPPLEMENTARY METHODS**

**Derivation of the glioma stem-like cells NCH397**

NCH397 cells were derived from fresh IDHwt glioblastoma tissue and propagated as described in detail earlier [1]. The study was approved by the institutional ethics committee and was conducted in accordance with the Declaration of Helsinki, with written consent from the patient undergoing tumor resection at the Department of Neurosurgery, Military University Hospital in Prague, Czech Republic. Briefly, the procedure was as follows: Macroscopic vessels and necrotic tissue was removed, the tissue was minced using a sterile scalpel. Afterwards, the fragments of tumor tissue were incubated with the Papain Dissociation System kit (Worthington Biochemical Corporation) according to the manufacturer’s protocol. After incubation, the papain was quenched by albumin-ovomucoid solution, debris and erythrocytes were removed by discontinuous gradient centrifugation and single cells were cultivated in Neurobasal Medium minus phenol red (Sigma), 1% Glutamax (Gibco), 1% penicillin-streptomycin (Sigma), 2% B-27™ Supplement (Gibco) and 20 ng/ml EGF and 20 ng/m FGF (PeproTech) in non-adherent cell culture flasks. After brief expansion, the Neurobasal Medium minus phenol red (Sigma) was changed to DMEM-F12 without phenol red (Sigma) and cells propagated further in the form of spheres (NHC397A cells), or in Geltrex (0.05 mg/ml; Thermo Fisher Scientific) coated flasks (NHC397AG cells).

**Generation of stable cell lines**

Vectors used in this study were constructed in the lab with standard molecular cloning procedures; details as well as the plasmids themselves are available upon request. Briefly, for stable cell lines expressing inducible EGFP-NAV3, the EGFP-NAV3 sequence was cut from pN1-EGFP-NAV3 vector (generous gift of prof. Yarden) and cloned into Sleeping Beauty donor vector using blunt ends. The pSB-NAV3 vector was co-transfected into cells along with Sleeping Beauty transposase (SB100X). For Dendra2 stable cell line, the Dendra2 fluorophore sequence was cloned into pIrespuro3 vector, which was linearized before transfection into cells. Populations of stably transfected cells were enriched for the respective encoded fluorescence with a cell sorter.

**Adhesion assay**

Cellular adhesion was analyzed by a standard Plate and Wash assay. Briefly, prior to the experiment, cells were starved for 3 hours, and coating of 96-well plates was prepared (100 µl per well) using collagen I (Millipore, 08-115), collagen IV (Cultrex, 3410-010-02), and fibronectin (Merck, F4759); concentrations were 1 µg/ml, 5 µg/ml, and 50 µg/ml in PBS. At least 8 technical replicates were prepared per condition. 1% BSA was used as a negative control. Before seeding the cells, plates were blocked with 1% BSA for 15 minutes, after which 50 000 cells per well (in 100 µl) were left to adhere for 30 min. Medium was then quickly decanted, whole plates were washed twice in PBS and 50 µl of Fix and Stain solution (0.1% Crystal violet in 20% methanol) was added per well followed by incubation at 4 °C over night. The next day, plates were washed intensively with water and 50 µl of 0.1% Triton X-100 was added to each well. After several hours of mild shaking, the absorbance was measured at 595 nm. Data from three independent biological experiments were combined and statistically evaluated in GraphPad Prism using unpaired t-tests.

**Proliferation assay**

Proliferative capacity of cells was assessed using the Alamar Blue assay according to manufacturer’s protocol. Briefly, the AlamarBlue reagent (Invitrogen) was added to cell culture medium (1:10) and incubated with cells for 1 hour. The medium containing AlamarBlue was then transferred to new wells and fluorescence (excitation 550 nm; emission 590 nm) was measured using the Infinite M200 Pro plate fluorimeter (TECAN). The results are summarized from 3 independent biological replicates and were statistically analyzed in GraphPad Prism using unpaired t-tests.

**Antibodies and inhibitors used in Supplementary results**

Primary antibodies used include: Ac-Tubulin (5335S, Cell Signaling), P-CAS Y410 (4011, Cell Signaling), P-Erk (4377, Cell Signaling), P-FAK Y861 (AJ1285f, Abgent), P-Stat3 Y705 (9138, Cell Signaling), P-PAX Y118 (2541, Cell Signaling), Integrin β1 (sc-46655, Santa Cruz Biotechnology), all diluted 1:1000 in 2% BSA in TTBS. Inhibitors used include 5 μM PF-562271 (Sigma) and 10 μM SB202190 (Santa Cruz Biotechnology).

1. Sana, J. *et al.* (2018) Identification of microRNAs differentially expressed in glioblastoma stem-like cells and their association with patient survival. *Sci. Rep.* 8, 2836

**SUPPLEMENTARY FIGURES AND LEGENDS**

**
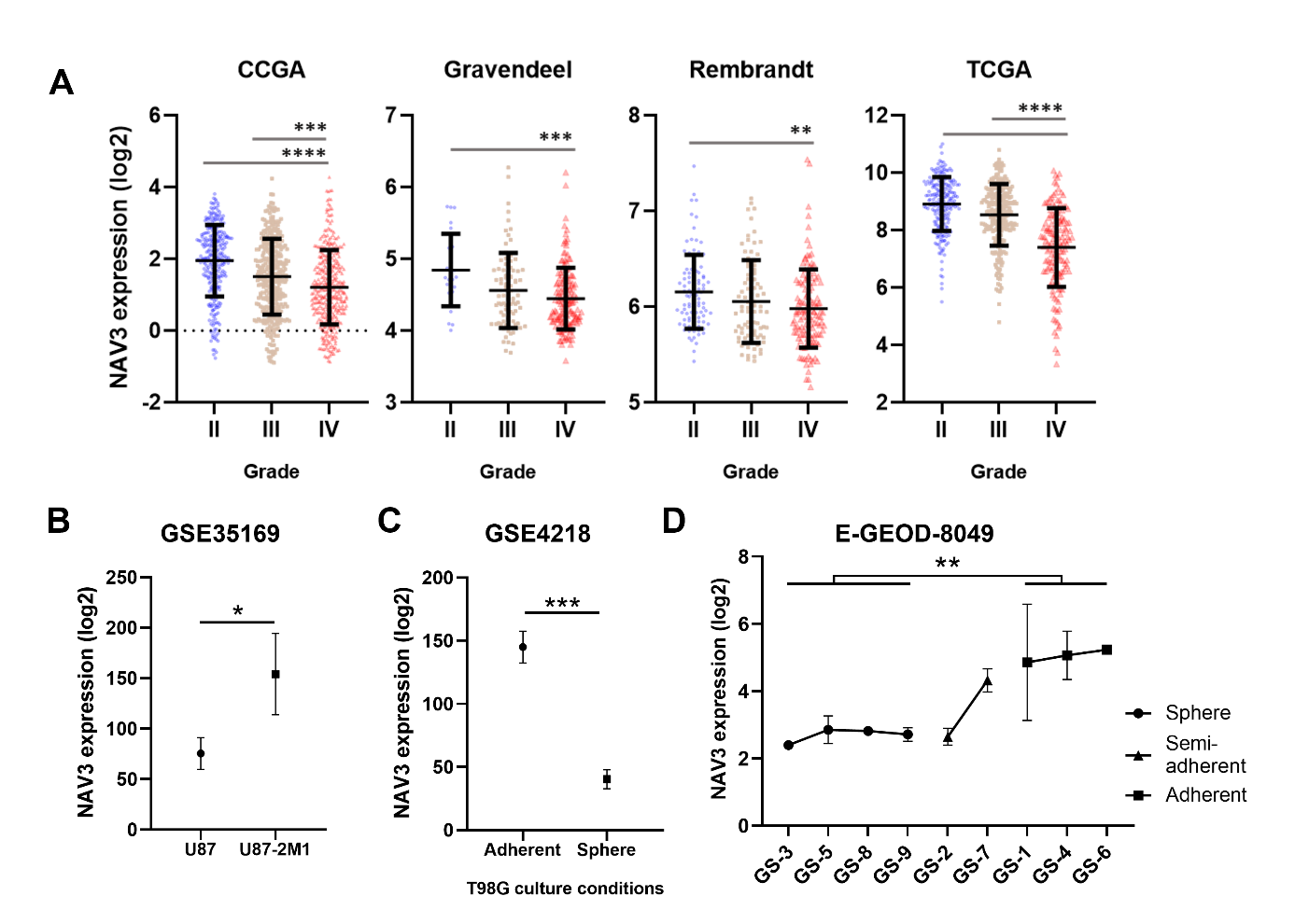
**

**Supplementary Figure S1: Additional data analysis of NAV3 expression in glioma.** A) NAV3 expression levels from CCGA, Gravendeel, Rembrandt and TCGA datasets according to tumor grade based on now outdated WHO 4^th^ edition classification. Statistical significance was determined using one-way ANOVA with post-hoc Tukey test. B) NAV3 expression from dataset GSE35169 (accessed using http://www.biogps.org) depicting NAV3 levels in parental GBM U87 cells and derived metastatic cell line U87-2M1. Statistical significance was determined using two-tailed t-test. C) NAV3 expression in the T98G GBM cell line according to culture conditions from dataset GSE4218 (accessed using https://www.ncbi.nlm.nih.gov/geo/). Statistical significance determined by two-tailed t-test. D) NAV3 expression from dataset E-GEOD-8049 (accessed using http://www.biogps.org) depicting NAV3 levels in individual GBM derived stem cell-like lines sorted according to culture growth pattern. Statistical significance determined by ANOVA with Tukey's multiple comparisons test. Data are shown as mean ±SD; *p ≤ 0.05, ** p ≤ 0.01, ***p ≤ 0.001, ****p ≤ 0.0001.

**
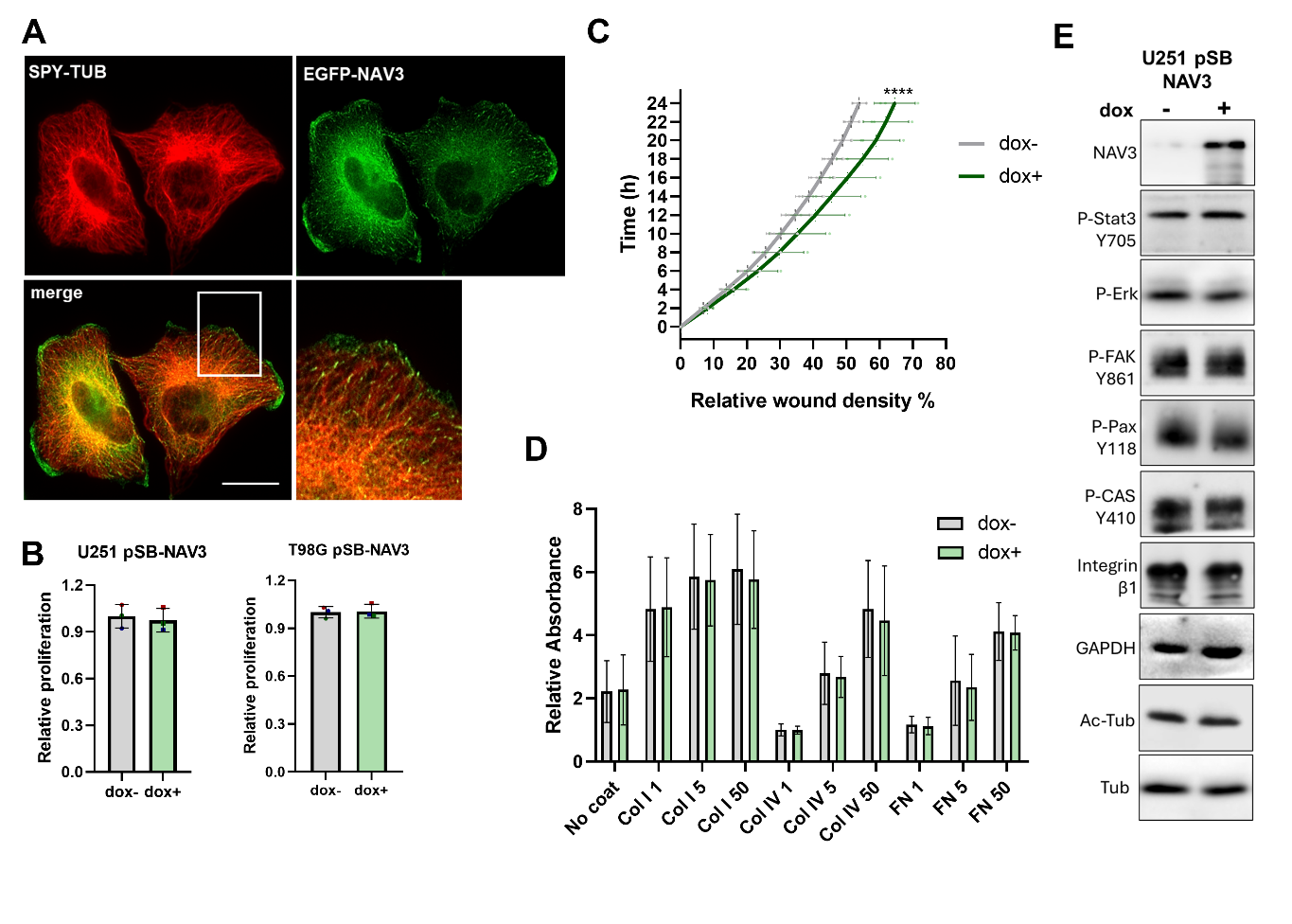
**

**Supplementary Figure S2: Additional data for U251 and T98G pSB-NAV3 cell lines.** A) Representative image of T98G pSB-NAV3 dox+ cell with immunofluorescently labeled MTs (red) depicting EGFP-NAV3 localization. Image inset shows localization of NAV3 to MT ends. Scale bar 25 µm. B) Results of Alamar Blue proliferation assay for both U251 and T98G pSB-NAV3. Differences non-significant. C) Results of 2D wound healing assay conducted using U251 pSB-NAV3 cells ± dox on uncoated surface plotted as increasing wound density during 24 h after scratch. Statistical significance was determined using ratio paired t-tests; ****p ≤ 0.0001. D) Analysis of adhesion assay using U251 pSB-NAV3 cells ± dox seeded on various coated surfaces; numbers at ECM proteins indicate concentrations in µg/ml. Differences non-significant. E) Immunoblotting of adhesion associated proteins in samples from U251 pSB-NAV3 ± dox cells. Representative image of three independent replicates is shown. Results shown in B-D are compiled from three independent replicates and presented as mean ±SD.

**
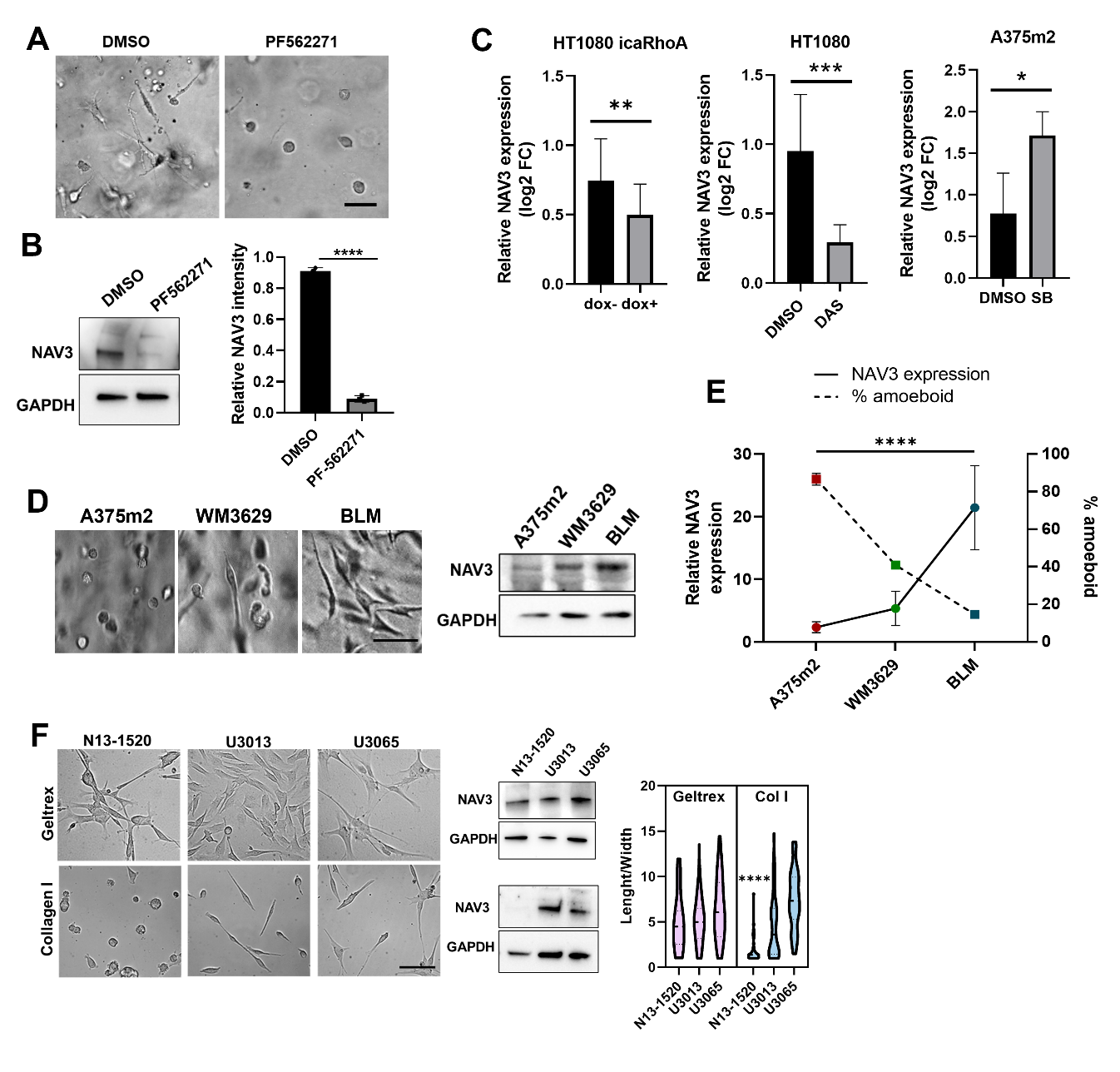
**

**Supplementary Figure S3: Additional data on the association between invasion plasticity and NAV3 expression.** A) Representative images of U251 cells treated with DMSO or 5 μM PF-562271 (FAK inhibitor). Scale bar 100 μm. B) Western blot detection of NAV3 in U251 cells treated with DMSO or PF-562271 for 24 h (left) and corresponding quantification based on two independent replicates. C) RT-qPCR validation of RNA-seq results (see Fig. 5) showing differential expression of NAV3 during MAT (fibrosarcoma cells HT1080 RhoA ±dox and HT1080 cells treated with either DMSO or 1 μM DAS) and AMT (A375m2 melanoma cells treated with either DMSO or 10 μM SB202190). Statistical significance of three independent replicates was determined using t-tests. D) Representative images of three melanoma cell lines embedded in 3D collagen (left) and according immunoblotting of NAV3 (right). Scale bar 100 μm. E) Quantification of melanoma cells‘ morphology in 3D collagen in relation to NAV3 expression determined by RT-qPCR. Results shown are based on three independent replicates. F) Representative images of patient-derived GSCs (N13-1520, U3013 and U3065) seeded on either Geltrex or collagen I coated surfaces, scale bar 100 μm (left). Corresponding western blot detection of NAV3 from cells on Geltrex or collagen I (middle) and quantification of cell morphology on either substrate measured as length/width ratio (right). Statistical significance of two independent replicates was determined using t-tests. *p ≤ 0.05, ** p ≤ 0.01, ***p ≤ 0.001, ****p ≤ 0.0001.


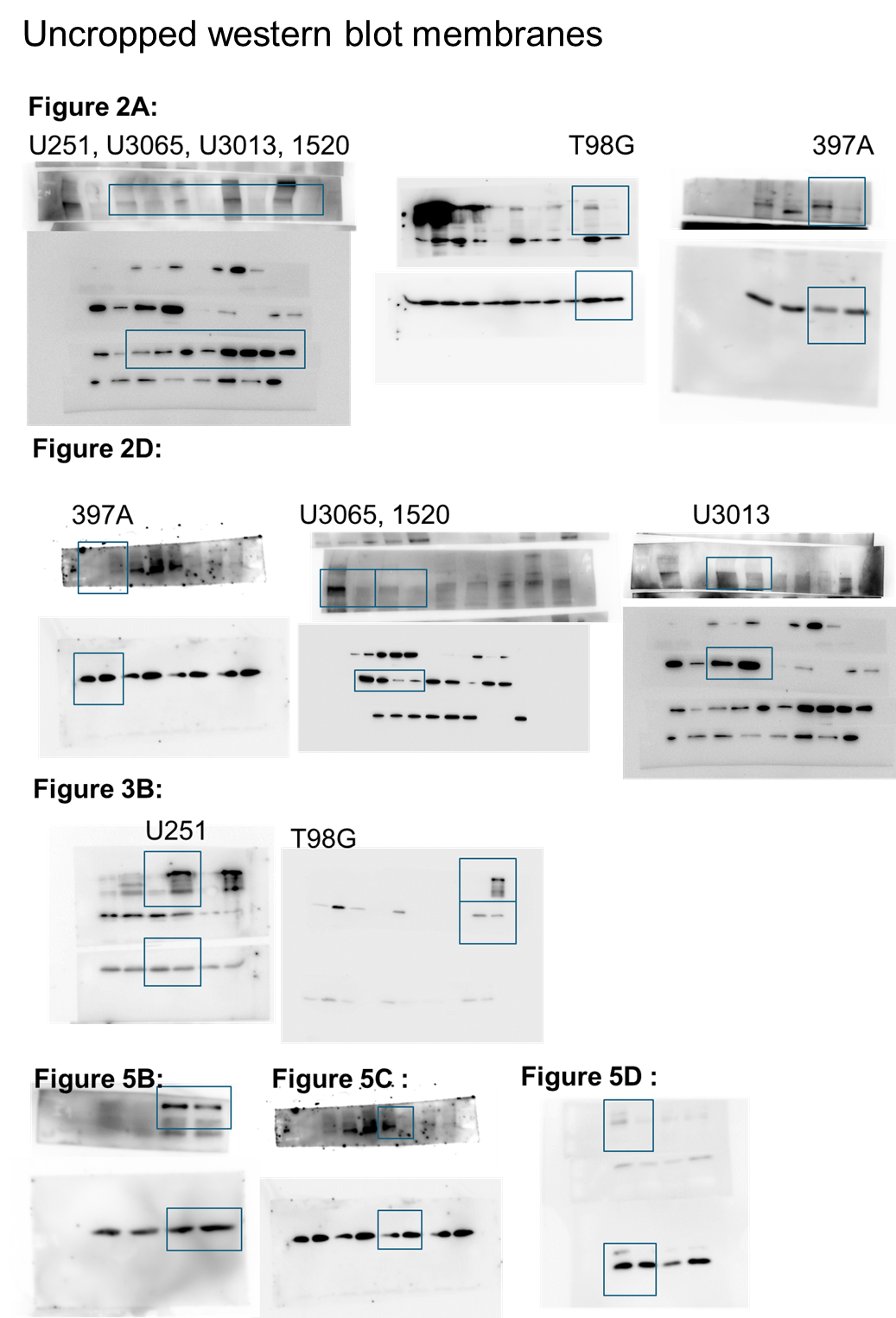


**Supplementary Figure S4:** Uncropped western blot membranes.
